# Effectiveness of pancreatitis activity score plus coagulation function and serum calcium in predicting the severity and outcome of acute pancreatitis

**DOI:** 10.12669/pjms.41.11.12085

**Published:** 2025-11

**Authors:** Xin Hu, Fang Xu, Zhan-biao Yu

**Affiliations:** 1 Xin Hu Electrocardiogram Room, Affiliated Hospital of Hebei University, Baoding, Hebei, 071000, P. R. China; 2 Fang Xu Department of ICU, Affiliated Hospital of Hebei University, Baoding, Hebei, 071000, P. R. China; 3 Zhan-biao Yu Department of ICU, Affiliated Hospital of Hebei University, Baoding, Hebei, 071000, P. R. China

**Keywords:** Acute pancreatitis, Coagulation factor, PASS score, Serum calcium, Severity

## Abstract

**Objective:**

To examine the relationship between acute pancreatitis (AP) severity and both coagulation function and serum calcium levels.

**Methods:**

This is a retrospective study. A total of one hundred patients with AP admitted to our hospital from July 2022 to January 2024. Fifty patients with severe AP(SAP) were assigned to the experimental group, fifty patients with mild-to-moderate AP were assigned to the control group. The pancreatitis activity score system(PASS) score, platelet(PLT) count, von Willebrand factor antigen(vWF:Ag), prothrombin time(PT), activated partial prothrombin time, fibrinogen, thrombin time, antithrombin III(ATIII), plasminogen, serum calcium(Ca) and D-dimer(D-D) were compared. The independent risk factors for predicting AP severity were analysed. The prognostic predictive value and diagnostic efficacy of these independent risk factors were assessed.

**Results:**

The experimental group exhibited a statistically significant increase in the PASS score, vWF:Ag, PT and D-D levels and a significant decrease in PLT, ATIII and Ca levels compared with the control group(P< 0.001). Logistic regression analysis showed that PASS, vWF:Ag, PT, ATIII, D-D and Ca were independent risk factors for predicting the severity of AP; Ca levels had the highest diagnostic efficacy, followed by ATIII and PT; vWF:Ag was the least effective. The levels of vWF:Ag, PT, and D-D are positively correlated with PASS scores, the levels of ATIII and Ca are negatively correlated.

**Conclusion:**

The levels of vWF:Ag, PT, D-D, ATIII, and Ca in AP patients are correlated with the severity of the condition, and Ca levels may more accurately and early assess the severity of AP patients.

## INTRODUCTION

Acute pancreatitis(AP) occurs because of the activation of pancreatic enzymes in the pancreas caused by various factors.^1^ A study revealed that the incidence of AP is about 1/1000 to 1/10000,^2^ with an overall mortality rate of 5%–10%. It has been observed that 20%–30% of AP cases progress to severe acute pancreatitis(SAP), with a mortality rate of 30%–50%.^3^ A study conducted by van Dijk SM et al. suggests that multiple-organ failure is the main cause of worsening or even death in patients with AP.^4^ Early and accurate assessment of the severity of patients with AP and active intervention and treatment measures are key to reducing mortality and improving prognosis. Although previous studies have proposed the establishment of scoring systems such as APACHE II and BISAP to assess the severity of the patient’s disease,^5^ the number of items and the complexity of scoring systems have limited their application.^6^

The pancreatitis activity score system(PASS) has been demonstrated to be of great importance in the evaluation of moderate and severe AP.^7^ In the case of AP, the inflammatory response not only activates platelets(PLTs) but also activate the coagulation system and disrupt the dynamic balance of the coagulation and anticoagulation systems.^8^ According to Peng et al.^9^ patients with SAP have decreased serum calcium levels and AP patients with a low calcium level show high mortality. Therefore, in this study, the effectiveness of integrating PASS with serum calcium and coagulation function to measure the prognosis and severity of AP was assessed.

## METHODS

This is a retrospective study. A total of 100 patients who were admitted to Affiliated Hospital of Hebei University from July 2022 to January 2024 were enrolled in study and classified into three groups based on the severity of condition: moderately severe acute pancreatitis (MSAP), SAP and mild acute pancreatitis (MAP). Fifty patients with SAP were assigned to the experimental group, whereas 50 patients with MSAP and MAP were assigned to the control group. Patient data including demographic data, diagnosis of Acute Pancreatitis, were retrieved from electronic medical record systems.

### Ethical Approval:

The study was approved by the Institutional Ethics Committee of Affiliated Hospital of Hebei University(No.: HDFYLL-KY-2023-009; date: January 19, 2023), and written informed consent was obtained from all participants.

### Inclusion criteria:

− Patients fulfil the diagnostic standard for AP.^10^
− Patients aged 70 years old or younger.
− Patients whose time interval from onset to admission was ≤24 h.
− Patients with complete clinical data.
− Patients were briefed on this study and who provided written consent.

### Exclusion criteria:

− Patients have a past medical history of pancreatic disease.
− Patients are experiencing recurrent pancreatitis.
− Patients have important organ diseases such as heart, lung, liver or kidney.
− Patients have a prior history of abnormal coagulation or haematologic disorders.
− Patients have taken medications that may recently influence the research results such as anticoagulants or PLT inhibitors.
− patients with neurological or psychiatric disorders or cognitive impairment that prevented them from cooperating to complete the study.

### Observation indexes and criteria:

All patients had 10 mL of elbow vein blood drawn upon admission, and the blood samples were centrifuged at 4000 r/min for 10 minutes. Using a Hitachi 7600 automatic biochemical analyser and matching reagents (Hitachi, Japan), patients’ coagulation-related parameters, including von Willebrand factor antigen (vWF:Ag), activated partial prothrombin time (APTT), platelet (PLT), plasminogen (PLG), thrombin time (TT), fibrinogen (FIB), prothrombin time (PT), antithrombin III (ATIII) and D-dimer (D-D), were measured using enzyme-linked immunosorbent assay, and their serum calcium (Ca) levels were measured using an integrated biochemical and immune automatic analyser.

The PASS scoring criteria: (1) organ failure (1 point for each of the respiratory, circulatory and renal systems, whilst cumulative for multiple-system failure) × 100; (2) intolerance to solid food (one point for intolerance, whilst 0 point for tolerance) × 40; (3) SIRS (one point for each abnormal diagnostic criterion, whilst cumulative for multiple abnormalities) × 25; (4) abdominal pain (0 to 10 points for the pain scoring scale) × 5; (5) analgesia by intravenous route (converted to the equivalent amount of 1 mg of morphine) × 5. Each of the abovementioned scores was multiplied by the number of weights following the item. Next, the five scores were summed to obtain the PASS score. The PASS score of >140 indicates moderate SAP and vice versa for MAP.^11^ Disease severity was assessed and categorised following the 2012 version of the Atlanta AP Classification Criteria.^12^ MAP indicates local or systemic complications or no organ failure; MSAP refers to the presence of local or systemic complications or transient organ failure (recovery within 48 h); SAP indicates relentless (>48 h) organ failure.

### Statistical analysis:

SPSS20.0 statistical software package was used for processing. The confidence interval was 95%. Measurement data were represented by(Mean±SD), and compared was performed by the independent sample t-test. Qualitative data was represented by n (%), and comparison was represented by chi square test. The influencing factors were analyzed using logistic regression analysis. Calculated the area under the curve (AUC) and the best diagnostic critical value for each index to predict SAP. Pearson correlation analysis was conducted to detect the correlation between the indexes and disease severity, *p*< 0.05 indicated a statistically significant difference.

## RESULTS

There was no significant difference in general data between the two groups(P>0.05), as shown in Table-I. The PASS score and vWF: Ag, PT, D-D levels in the research group were higher than those in the control group, while PLT, ATIII, and Ca levels were significantly lower than those in the control group, with statistical significance (p=0.00). However, there was no significant difference in other indicators between the two groups (*P* > 0.05) (Table II).

**Table-I:** Comparative analysis of the general data of both groups (*χ̅*±*S*); n = 50.

Indexes	Experimental group	Control group	t	P
Male (cases, %)	25 (50%)	23 (46%)	0.16	0.69*
Age (years)	55.62 ± 11.77	53.60 ± 11.52	0.87	0.39^△^
Course of the disease (h)	18.64 ± 1.56	18.76 ± 1.72	0.37	0.72^△^
BMI (kg/m^2^)	23.95 ± 2.61	23.22 ± 2.64	1.40	0.16^△^
Aetiology				
Biliogenic aetiology (cases, %)	27 (54%)	25 (50%)	0.16	0.69*
Alcoholic aetiology (cases, %)	19 (38%)	18 (36%)	0.04	0.84*
Other (cases, %)	4 (8%)	7 (14%)	0.92	0.34*

***Note:***

* c^2^ test,

△ independent sample t-test

**Table-II:** Analysis of the differences in PASS score and laboratory tests between the two groups (*χ̅*±*S*); n = 50.

Indexes	Experimental group	Control group	t	P^△^
PASS score*	144.60 ± 13.28	119.20 ± 7.58	11.74	<0.001
vWF:Ag (%)*	326.82 ± 10.06	278.93 ± 12.42	21.19	<0.001
PLT (10^9^/L)*	125.28 ± 8.20	174.44 ± 5.09	36.01	<0.001
PT (S)*	15.73 ± 2.48	12.13 ± 2.53	7.16	<0.001
APTT (S)	31.44 ± 2.51	30.50 ± 2.59	1.85	0.07
TT (S)	18.30 ± 2.36	17.49 ± 2.49	1.66	0.10
FIB (g/L)	3.96 ± 2.01	3.90 ± 1.75	0.16	0.87
ATIII (%)*	69.10 ± 4.95	75.70 ± 4.58	6.92	<0.001
PLG (%)	105.48 ± 5.35	107.35 ± 5.66	1.70	0.09
D-D(mg/L)*	12.60 ± 1.59	7.77 ± 1.76	14.44	<0.001
Ca(pg/mL)*	1.78 ± 0.36	2.54 ± 0.31	11.23	<0.001

***Note:***

△ independent sample t-test

Multifactorial logistic regression analysis indicated that PASS (*P* < 0.001), vWF:Ag (*P* < 0.001), PT(*P*< 0.001), ATIII(*P*< 0.001), D-D (*P* < 0.001) and AMD Ca (*P* < 0.001) were independent risk factors for predicting the severity of AP (Table III). The AUCs of PASS score, vWF:Ag, PT, ATIII, D-D and Ca were 4.9%, 0.5%, 16.2%, 82.6%, 2.2% and 93.9%, respectively(Table-IV). Correlation analysis indicated that PASS score is positively correlated with vWF: Ag, PT, and D-D levels, while negatively correlated with ATIII and Ca levels (Table-V).

**Table-III:** Logistic regression analysis of the severity of acute pancreatitis of independent risk factors (*χ̅*±*S*); n = 50.

Indexes	β value	SE value	Waldχ^2^	P value	95.0%CI
PASS*	−0.262	0.060	19.290	0.000	0.685–0.865
vWF:Ag*	−0.170	0.034	25.451	0.000	0.789–0.901
PLT	1.135	174.871	0.000	0.995	0.000–2.20
PT*	−0.557	0.113	24.250	0.000	0.459–0.715
APTT	−0.147	0.084	3.296	0.069	0.736–1.012
TT	−0.138	0.084	2.680	0.102	0.738–1.028
FIB	−0.017	0.107	0.026	0.872	0.796–1.213
ATIII*	0.270	0.055	24.392	0.000	1.177–1.458
PLG	0.062	0.037	2.817	0.093	0.990–1.145
D-D*	−1.425	0.278	26.353	0.000	0.140–0.414
Ca*	7.725	1.823	17.953	0.000	63.536–80681.004

^*^ *P* < 0.05.

**Table-IV:** Diagnostic efficacy of independent risk factors for acute pancreatitis (*χ̅*±*S*); n = 50.

Indexes	Critical value	Sensitivity %	Specificity %	AUC	Youden index	95.0%CI	P value
PASS score	127.50	0.160	0.120	0.049	0.720	0.013–0.085	0.000
vWF:Ag (%)	312.55	0.020	0.040	0.005	0.940	0.000–1.000	0.000
PT (S)	15.35	0.080	0.400	0.162	0.520	0.087–0.237	0.000
ATIII (%)	67.80	1.000	0.540	0.826	0.540	0.747–0.905	0.000
D-D (mg/L)	10.15	0.100	0.060	0.022	0.840	0.000–0.043	0.000
Ca (pg/mL)	2.15	0.860	0.860	0.939	0.720	0.898–0.981	0.000

**Table-V:** Correlation analysis of independent risk factors such as PASS score, coagulation factors and blood calcium (r).

	PASS score		vWF:Ag (%)		PT (S)			ATIII (%)			D-D (mg/L)			Ca (pg/mL)	
	r value	P value	r value	P value	r value	P value	r value		P value	r value		P value	r value		P value
Acute pancreatitis	−0.765	0.000	–0.906	0.00	–0.586	0.000	0.573		0.000	–0.825		0.000	0.750		0.000

## DISCUSSION

As confirmed by the results of the present study, coagulation dysfunction is evident in AP, and the relevant laboratory indicators can be used to predict the progression of the disease at an early stage.^13^ In this study, PASS score, vWF:Ag, PT and D-D were substantially increased compared with the control group, whereas PLT, ATIII and Ca revealed considerable decreases (*P* < 0.001). Moreover, PASS (*P* < 0.001) and vWF were indicated by multi-factor logistic regression analysis results: Ag (*P* < 0.001), PT (*P* < 0.001), ATIII (*P* < 0.001), D-D (*P* < 0.001) and Ca (*P* < 0.001) were independent risk factors predicting the severity of AP. This result indicates the important role played by early severe endothelial injury in disease progression and prognosis. PLTs in the body form PLT-neutrophil complexes and adhere to endothelial cells under the combined effect of the damaged vascular endothelium and inflammation,^14^,^15^ leading to excessive PLT depletion and microthrombus formation. The presence of PLT overconsumption and activation in patients with AP has been described in the literature, and the level of PLT at admission correlates with patient prognosis.^16^ In the present study, patients with severe AP had significantly lower PLT levels compared with patients with mild-to-moderate disease.

The abnormal activation of PLTs may lead to microthrombosis, thereby causing microcirculatory disorders and further stress damage to the vascular endothelium.^17^ In this study, patients with SAP had a significant increase in coagulation factor depletion, as evidenced by the prolongation of PT time and a decrease in ATIII levels. These results correlate with the severity of the disease. In the study conducted by Fan et al. for every 1% decrease in the value of ATIII change,^18^ the patient had a 0.12-fold increase in the risk of possible death. D-dimer the more severe the disease, the more pronounced the elevation. As demonstrated in this study, an imbalance of the coagulation and activation of the anticoagulation/fibrinolytic system is found in patients with severe pancreatitis.

Calcium ions, as coagulation factors, are heavily depleted with AP hypercoagulable-state coagulation factor depletion, resulting in severe hypocalcaemia.^19^ According to Liu et al.,^20^ the predictive value of serum calcium for AP in persistent organ failure. In our study, serum Ca levels were significantly lower in the experimental group than in the control group (*P* = 0.00). Multifactorial logistic regression suggested that Ca (*P* = 0.000) was an independent risk factor for predicting the severity of AP, and it had the highest diagnostic efficacy with an AUC of 93.9%. Moreover, Ca levels were positively correlated with the severity of the disease.

Our research confirmed that patients with SAP in the experimental group had considerably more eminent PASS scores than the control group(*P*= 0.00), and multifactorial logistic regression analysis demonstrated that the PASS score was an independent risk factor for predicting AP severity(*P*= 0.000). Despite their importance to the prognostic assessment of AP, various existing scoring systems have complex operations with low specificity and sensitivity.^21^ The PASS can be used to assess the prognosis and severity of moderate and severe AP.^22^ The PASS can also be used to dynamically assess the prognosis of AP, and it has some clinical applications for moderate-to-severe AP.^23^

AP has a close correlation with serum calcium levels and coagulation function with regard to its severity. In general, patients with AP have increased levels of vWF:Ag, PT and D-D as well as decreased levels of ATIII and Ca, which correlate with the severity of the disease and serve as indicators for the determination and prognostic evaluation of the disease. In particular, Ca can be used to early and accurately evaluate the severity of AP in patients. In general, patients with AP have increased vWF:Ag, PT and D-D levels as well as decreased ATIII and Ca levels, which correlate with the severity of the disease and can be used as indicators for the determination and prognostic evaluation of the disease. In particular, the levels of Ca can assist in the prompt and precise assessment of the degree of AP severity.

### Limitations:

This study has a small sample size and a single-center design and retrospective in nature; thus, biases may occur. In future clinical work, the sample size will be further increased, and multi-centre and prospective cohort study methods will be included to explore the early diagnosis of AP in a more objective and detailed way so that more patients can benefit from it.

## CONCLUSION

This study confirms that serum calcium, AT III, PT, and D-D are independent risk factors for predicting the severity of acute pancreatitis, with serum calcium having the highest diagnostic efficacy. PASS scores have a synergistic effect with these indicators, but their predictive value is limited when applied alone.

### Authors’ Contributions:

**FX:** Carried out the studies, participated in collecting data, and drafted the manuscript, and are responsible and accountable for the accuracy or integrity of the work

**XH:** Performed the statistical analysis and participated in its design.

**ZBY:** Participated in acquisition, analysis, or interpretation of data and draft the manuscript.

All authors have read and approved the final manuscript.

## References

[1] Garg PK, Singh VP (2019). Organ Failure Due to Systemic Injury in Acute Pancreatitis. Gastroenterology..

[2] James TW, Crockett SD (2018). Management of acute pancreatitis in the first 72 hours. Curr Opin Gastroenterol..

[3] Mederos MA, Reber HA, Girgis MD (2021). Acute pancreatitis:a review. JAMA..

[4] Van Dijk SM, Hallensleben NDL, van Santvoort HC, Fockens P, van Goor H, Bruno MJ (2017). Acute pancreatitis:recent advances through randomised trials. Gut..

[5] Arif A, Jaleel F, Rashid K (2019). Accuracy of BISAP score in prediction of severe acute pancreatitis. Pak J Med Sci..

[6] Gliem N, Ammer-Herrmenau C, Ellenrieder V, Neesse A (2021). Management of severe acute pancreatitis:an update. Digestion..

[7] Buxbaum J, Quezada M, Chong B, Gupta N, Yu CY, Lane C (2018). The Pancreatitis Activity Scoring System predicts clinical outcomes in acute pancreatitis:findings from a prospective cohort study. Am J Gastroenterol..

[8] Li ZF, Xu MY, Zhang DH, Gao TT, Gao Z, Li H (2020). Effects of ulinastatin combined with octreotide on blood coagulation function, inflammatory factors and amylase in patients with severe acute pancreatitis. J Biol Regul Homeost Agents..

[9] Peng T, Peng X, Huang M, Cui J, Zhang Y, Wu H (2017). Serum calcium as an indicator of persistent organ failure in acute pancreatitis. Am J Emerg Med..

[10] Banks PA, Bollen TL, Dervenis C, Gooszen HG, Johnson CD, Sarr MG (2013). Classification of acute pancreatitis--2012:revision of the Atlanta classification and definitions by international consensus. Gut..

[11] Knoph CS, Cook ME, Fjelsted CA, Novovic S, Mortensen MB, Nielsen LBJ (2021). Effects of the peripherally acting μ-opioid receptor antagonist methylnaltrexone on acute pancreatitis severity:study protocol for a multicentre double-blind randomised placebo-controlled interventional trial, the PAMORA-AP trial. Trials..

[12] Banks PA, Bollen TL, Dervenis C, Gooszen HG, Johnson CD, Sarr MG (2013). Classification of acute pancreatitis--2012:revision of the Atlanta classification and definitions by international consensus. Gut..

[13] Olson E, Perelman A, Birk JW (2019). Acute management of pancreatitis:the key to best outcomes. Postgrad Med J..

[14] Nielsen L, Holm J, Rozanski E, Meola D, Price LL, De Laforcade A (2019). Multicenter investigation of hemostatic dysfunction in 15 dogs with acute pancreatitis. J Vet Emerg Crit Care (San Antonio)..

[15] Maduzia D, Ceranowicz P, Cieszkowski J, Chmura A, Galazka K, Kusnierz-Cabala B (2020). Administration of warfarin accelerates the recovery in ischemia/reperfusion-induced acute pancreatitis. J Physiol Pharmacol..

[16] Yarkaç A, Kose A, Bozkurt BabuşS, Ates F, Orekici Temel G, Ölmez A (2019). The value of hematological parameters in acute pancreatitis. Ulus Travma Acil Cerrahi Derg..

[17] Wollny T, Wątek M, Wnorowska U, Piktel E, Góźdź S, Kurek K (2022). Hypogelsolinemia and decrease in blood plasma sphingosine-1-phosphate in patients diagnosed with severe acute pancreatitis. Dig Dis Sci..

[18] Fan C, Song Y, Wang X, Mao C, Xiong Y (2022). Identification of early derangements of coagulation, hematological and biochemical profiles in patients with acute pancreatitis. Clin Biochem..

[19] Yuan S, Giovannucci EL, Larsson SC (2021). Gallstone disease, diabetes, calcium, triglycerides, smoking and alcohol consumption and pancreatitis risk:Mendelian randomization study. NPJ Genom Med.

[20] Liu B, Li D, Hu X, Cao Y (2018). The predictive value of serum calcium for acute pancreatitis with persistent organ failure. Am J Emerg Med.

[21] Yano T, Taniguchi M, Shirasaka T, Tsuneyoshi I (2019). Effectiveness of soluble recombinant human thrombomodulin in patients with severe acute pancreatitis complicated by disseminated intravascular coagulation. Turk J Anaesthesiol Reanim..

[22] Paragomi P, Hinton A, Pothoulakis I, Talukdar R, Kochhar R, Goenka MK (2022). the modified pancreatitis activity scoring system shows distinct trajectories in acute pancreatitis:an international study. Clin Gastroenterol Hepatol..

[23] Yu Z, Ni Q, Zhang P, Jia H, Yang F, Gao H (2022). Clinical utility of the pancreatitis activity scoring system in severe acute pancreatitis. Front Physiol..

